# The sociodemographic and clinical profile of patients with major depressive disorder receiving SSRIs as first-line antidepressant treatment in European countries

**DOI:** 10.1007/s00406-021-01368-3

**Published:** 2022-01-06

**Authors:** Gernot Fugger, Lucie Bartova, Chiara Fabbri, Giuseppe Fanelli, Markus Dold, Marleen Margret Mignon Swoboda, Alexander Kautzky, Joseph Zohar, Daniel Souery, Julien Mendlewicz, Stuart Montgomery, Dan Rujescu, Alessandro Serretti, Siegfried Kasper

**Affiliations:** 1 Department of Psychiatry and Psychotherapy, Medical University of Vienna, Vienna, Austria; 2 Department of Biomedical and NeuroMotor Sciences, University of Bologna, Bologna, Italy; 3 Social, Genetic and Developmental Psychiatry Centre, Institute of Psychiatry, Psychology and Neuroscience, King’s College London, London, UK; 4 Department of Human Genetics, Radboud University Medical Center, Donders Institute for Brain, Cognition and Behaviour, Nijmegen, The Netherlands; 5 Psychiatric Division, Chaim Sheba Medical Center, Tel Hashomer, Ramat Gan, Israel; 6 School of Medicine, Free University of Brussels, Brussels, Belgium; 7 Psy Pluriel-European Centre of Psychological Medicine, Brussels, Belgium; 8 Imperial College School of Medicine, University of London, London, UK; 9 Center for Brain Research, Medical University of Vienna, Spitalgasse 4, 1090 Vienna, Austria

**Keywords:** Major depressive disorder, Antidepressant treatment, Antidepressants, Selective-serotonin reuptake inhibitors

## Abstract

**Introduction:**

Due to favorable antidepressant (AD) efficacy and tolerability, selective-serotonin reuptake inhibitors (SSRIs) are consistently recommended as substances of first choice for the treatment of major depressive disorder (MDD) in international guidelines. However, little is known about the real-world clinical correlates of patients primarily prescribed SSRIs in contrast to those receiving alternative first-line ADs.

**Methods:**

These secondary analyses are based on a naturalistic, multinational cross-sectional study conducted by the European Group for the Study of Resistant Depression at ten research sites. We compared the socio-demographic and clinical characteristics of 1410 patients with primary MDD, who were either prescribed SSRIs or alternative substances as first-line AD treatment, using chi-squared tests, analyses of covariance, and logistic regression analyses.

**Results:**

SSRIs were prescribed in 52.1% of MDD patients who showed lower odds for unemployment, current severity of depressive symptoms, melancholic features, suicidality, as well as current inpatient treatment compared to patients receiving alternative first-line ADs. Furthermore, patients prescribed SSRIs less likely received add-on therapies including AD combination and augmentation with antipsychotics, and exhibited a trend towards higher response rates.

**Conclusion:**

A more favorable socio-demographic and clinical profile associated with SSRIs in contrast to alternative first-line ADs may have guided European psychiatrists’ treatment choice for SSRIs, rather than any relevant pharmacological differences in mechanisms of action of the investigated ADs. Our results must be cautiously interpreted in light of predictable biases resulting from the open treatment selection, the possible allocation of less severely ill patients to SSRIs as well as the cross-sectional study design that does not allow to ascertain any causal conclusions.

## Introduction

Selective-serotonin reuptake inhibitors (SSRIs) represent a very well studied class of antidepressant (AD) medication that is consistently recommended as the first-line psychopharmacotherapy for major depressive disorder (MDD) in clinical practice guidelines (CPG) throughout the world [25]. Accordingly, it is not surprising that SSRIs are the most commonly prescribed ADs in numerous patient populations with different ethnical and geographical backgrounds [2, 21, 27, 29, 46].

Despite the frequency of SSRIs prescriptions, rather heterogeneous conclusions were drawn in terms of efficacy. While SSRIs, particularly sertraline and escitalopram, were meta-analytically evidenced as gold standard ADs in MDD [12], other publications reported no substantial difference between SSRIs and other ADs [26]. The largest and most recent network meta-analysis (NMA) comprising over 116,000 patients reported rather small differences between the 21 investigated ADs that all performed better than placebo [11]. The SSRIs escitalopram and paroxetine, along with agents with a different mode of action, were, however, among the most efficacious, whereas the opposite was found for fluoxetine and fluvoxamine. Acceptability in that study was comparable between the studied ADs. As for individual SSRIs, escitalopram, citalopram, sertraline, and fluoxetine exhibited the lowest drop-out rates, while fluvoxamine the highest [11].

The efficacy and acceptability of SSRIs in MDD treatment may be only two of the important aspects influencing treatment choice. In spite of potential adverse effects (AE) that are most frequently related to the gastrointestinal tract and sexual dysfunction [10], SSRIs were repeatedly suggested to be less toxic in overdose and thus safer when compared to other drugs including the most ADs, especially tricyclic ADs (TCAs), but also more recently introduced AD substances as serotonin and norepinephrine reuptake inhibitors (SNRIs) and the noradrenergic and specific serotonergic AD (NaSSA) mirtazapine [31, 56]. Furthermore, previous studies underscored that SSRIs exhibit beneficial effects on life quality [36], cognitive functioning [63], and reduce relapse risk [13].

Although valuable knowledge on efficacy, tolerability and further important aspects distinguishing SSRIs from other ADs in MDD treatment could be retrieved from randomized-controlled trials (RCTs) and meta-analyses, extrapolation to real-world conditions found in everyday clinical practice is challenging, because of the manifold presentation of MDD which may vary in severity and course and which may include suicidality, psychotic-, melancholic- and atypical features, and/or comorbidities. Therefore, factors that may mediate clinicians’ choices to administer SSRIs over other ADs as first-line ADs in MDD in real-world settings are of interest. To shed light on these aspects, we aimed to illustrate the real-world prescription rates of SSRIs and other AD substances administered as first-line psychopharmacotherapy in 1410 MDD patients from Europe and Israel, and to reveal possible differences in their socio-demographic and clinical patterns.

## Materials and methods

### Study concept

The current analyses refer to the international and multicentric, observational, cross-sectional and non-interventional study with retrospective assessment of treatment response conducted by the European Group for the Study of Resistant Depression (GSRD) [3]. These secondary analyses are based on the GSRD project “Clinical and biological correlates of resistant depression and related phenotypes” performed from 2011 to 2016 at ten research centers (Vienna, Brussels, Toulouse, Elancourt, Halle, Athens, Bologna, Siena, Geneva and Tel Hashomer) in seven European countries and Israel. The design and all procedures of the GSRD study, which were introduced and comprehensively described in our previous reports [3], were approved by the ethics committees of each participating site.

### Study collective

Adult in- and outpatients of both sexes were recruited in academic as well as non-academic clinical routine centers in Austria, Belgium, France, Germany, Greece, Italy, Switzerland, and Israel that are mentioned above. In case of interest and eligibility for participation in the present study, all related procedures were thoroughly explained to the patients before signing written informed consent. To be included in the present study, the patients had to be diagnosed with a current single or recurrent major depressive episode (MDE) occurring in the course of MDD as their primary psychiatric diagnosis. Furthermore, they had to undergo an adequate psychopharmacotherapy with at least one AD agent employed in sufficient daily doses and treatment duration of minimally four weeks during the current MDE [3, 19]. The exclusion criteria comprised any primary psychiatric diagnosis other than MDD and co-occurring severe personality disorders and/or substance use disorders (except nicotine and/or caffeine) present six months before study enrollment. Other psychiatric and/or somatic comorbidities and potential additional features as psychotic and/or melancholic features and/or suicidality occurring during the current MDE were not excluded to ensure the naturalistic real-world conditions [3].

### Clinical assessment

To assure a high standard of data quality and inter-rater reliability, exclusively experienced psychiatrists undergoing specific rater trainings were allowed to assess socio-demographic, clinical, and treatment patterns of the enrolled MDD patients who were treated by senior consultants for psychiatry in all recruiting centers. In the course of a comprehensive clinical assessment, medical records of the included MDD patients were considered and the Mini International Neuropsychiatric Interview (MINI) [68] was applied to establish the primary psychiatric diagnosis according to the DSM-IV-TR criteria [73], the presence of potential additional specific features occurring during the current MDE, and/or psychiatric comorbidities. Furthermore, all administered treatments during the current MDE including the first-line AD treatment representing the initial AD agent were thoroughly assessed at study entry and documented accordingly. To measure depressive symptoms at study entry, reflecting a time-point after at least four weeks of adequate AD treatment, the 21-item Hamilton Rating Scale for Depression (HAM-D) [30] and the Montgomery and Åsberg Depression Rating Scale (MADRS; current MADRS, cMADRS) [58] were employed. To estimate the extent of depressive symptoms at the onset of the current MDE, reflecting a time-point prior to initiation of the first-line AD treatment that was minimally four weeks before study inclusion, the so-called retrospective MADRS (rMADRS) was calculated based on the MDD patients’ assertions and their medical records.

To determine treatment response patterns based on the GSRD staging model [3], the MADRS total score change (rMADRS–cMADRS) was calculated after at least one adequate AD trial administered during the current MDE. Accordingly, treatment response was defined by a cMADRS total score of < 22 and a ≥ 50% reduction of the MADRS total score after an AD trial of adequate daily dosing and duration lasting at least four weeks. A total cMADRS score of ≥ 22 and a < 50% MADRS total score reduction after one adequate AD trial was mandatory to categorize treatment non-response. Treatment resistance was determined as a non-response to two or more consecutive AD trials that were each administered in adequate daily dosing and duration during the current MDE [3].

In analogy to existing evidence, the current suicidal risk and its extent were ascertained according to the HAM-D item 3 that specifically assesses suicidality [17, 42]. Where applicable, low suicidality levels were reflected by the item-score of 1, while moderate to high degree of the current suicidal risk was depicted by the item-scores 2–4.

### Statistical computations

The enrolled 1410 patients suffering from primary MDD [3] were subdivided into two groups according to their first-line AD treatment with either SSRIs or alternative AD substances that was administered during the current MDE. Hereby, their socio-demographic and clinical patterns were represented with descriptive statistics (means, standard deviation (SD), and/or percentages) and, subsequently, dichotomously compared (Table 1). The initial analyses included chi-squared tests and analyses of covariance (ANCOVAs) for categorical and continuous variables, respectively. For ANCOVAs, the prescribed first-line AD treatment was included as fixed effect and recruitment center as covariate. The Bonferroni correction for multiple comparisons was applied in our initial analyses, whereby the alpha level, that was originally set at < 0.05, was further adjusted according to the number of variables tested (*n* = 50). Accordingly, the alpha level was set at = 0.001 (0.05/50 = 0.001). Uncorrected *p* values are displayed in Table 1, whereby statistical significance after the abovementioned correction for multiple tests is indicated in bold. Binary logistic regression analyses with the relevant independent variables withstanding the Bonferroni correction for multiple comparisons in our initial analyses were performed post hoc to quantify their association with the respective first-line AD treatment that represented the dichotomous dependent variable. The recruitment center served as covariate in the post hoc binary logistic regression analyses (Table 2), whereby the Bonferroni correction for multiple comparisons was applied in analogy to our initial analyses (*α* = 0.001). Data were analyzed employing the version 27 of IBM SPSS Statistics.

**Table 1:** Socio-demographic and clinical patterns of the GSRD patients who received first-line AD treatment with either SSRIs or other ADs during their current MDE

MDD patients’ charactersistics	Total sample (*n* = 1410)	SSRIs as first-line AD treatment (*n* = 734)	Other first-line AD treatment (*n* = 676)	*x*^2^/*F*	*p* value (ANCOVA/*x*^2^)
Sex, *n* (%)					
Female	943 (66.9)	501 (68.3)	442 (65.4)	1.310	0.252
Male	467 (33.1)	233 (31.7)	234 (34.6)		
Age, mean (SD), years (*n* = 1404)	50.3 (14.1)	50.1 (14.3)	50.4 (13.9)	0.514	0.473
Bodyweight, mean (SD), kilograms (*n* = 1387)	73.2 (16.8)	71.9 (16.7)	74.7 (16.8)	5.551	0.019
Ethnicity, *n* (%)					
Caucasian origin	1356 (96.2)	708 (96.5)	648 (95.9)	0.344	0.558
Education, *n* (%) (*n* = 1395)					
University education/non-university high education/high level general education	755 (54.1)	406 (55.9)	349 (52.2)	1.978	0.160
General secondary/technical education/elementary school/none	640 (45.9)	320 (44.1)	320 (47.8)		
Occupation, *n* (%) (*n* = 1408)					
Employed	659 (46.8)	378 (51.6)	281 (41.6)	13.943	**< 0.001**
Unemployed	749 (53.2)	355 (48.4)	394 (58.4)		
Relationship, *n* (%)					
Ongoing relationship	703 (49.9)	363 (49.5)	340 (50.3)	0.100	0.752
No ongoing relationship	707 (50.1)	371 (50.5)	336 (49.7)		
Disease course, *n* (%)					
Single MDE	127 (9.0)	69 (9.4)	58 (8.6)	0.289	0.591
Recurrent MDD	1283 (91.0)	665 (90.6)	618 (91.4)		
Number of MDEs during lifetime, mean (SD) (*n* = 1044)	3.3 (2.5)	3.2 (2.5)	3.4 (2.4)	0.526	0.468
Age of disease onset, mean (SD), years (*n* = 1329)	37.2 (15.4)	38.0 (15.6)	36.4 (15.3)	1.862	0.173
Duration of psychiatric hospitalizations during lifetime, mean (SD), weeks (*n* = 1328)	5.6 (20.5)	3.8 (13.7)	7.6 (25.7)	6.361	0.012
Additional features during the current MDE, *n* (%)					
Psychotic features	154 (10.9)	72 (9.8)	82 (12.1)	1.948	0.163
Melancholic features	856 (60.7)	400 (54.5)	456 (67.5)	24.778	**< 0.001**
Atypical features	33 (2.3)	15 (2.0)	18 (2.7)	0.590	0.442
Catatonic features	7 (0.5)	1 (0.1)	6 (0.9)	4.021	0.045
Suicidality^a^					
Current suicidal risk (dichotomous)	649 (46.0)	294 (40.1)	355 (52.5)	21.993	**< 0.001**
High/moderate level of suicidality	377 (58.1)	163 (55.4)	214 (60.3)	1.547	0.214
Low level of suicidality	272 (41.9)	131 (44.6)	141 (39.7)		
Treatment setting, *n* (%)					
Inpatient	488 (34.6)	185 (25.2)	303 (44.8)	59.845	**< 0.001**
Outpatient	922 (65.4)	549 (74.8)	373 (55.2)		
Duration of the current MDE, mean (SD), days (* n* = 1114)	204.7 (164.6)	200.8 (150.7)	208.8 (177.9)	1.907	0.168
Psychiatric comorbidities, *n* (%)					
Any anxiety disorder	294 (20.9)	161 (21.9)	133 (19.7)	1.089	0.297
Generalized anxiety disorder	151 (10.7)	88 (12.0)	63 (9.3)	2.623	0.105
Panic disorder	114 (8.1)	64 (8.7)	50 (7.4)	0.829	0.363
Agoraphobia	113 (8.0)	51 (6.9)	62 (9.2)	2.360	0.125
Social phobia	45 (3.2)	21 (2.9)	24 (3.6)	0.541	0.462
Obsessive–compulsive disorder (*n* = 1397)	22 (1.6)	14 (1.9)	8 (1.2)	1.190	0.275
Posttraumatic stress disorder	20 (1.4)	7 (1.0)	13 (1.9)	2.365	0.124
Somatic comorbidities, *n* (%)					
Any somatic comorbidity	653 (46.3)	323 (44.0)	330 (48.8)	3.276	0.070
Hypertension	267 (18.9)	130 (17.7)	137 (20.3)	1.497	0.221
Thyroid dysfunction	204 (14.5)	102 (13.9)	102 (15.1)	0.404	0.525
Migraine	156 (11.1)	75 (10.2)	81 (12.0)	1.113	0.291
Diabetes	84 (6.0)	34 (4.6)	50 (7.4)	4.800	0.028
Heart disease	72 (5.1)	37 (5.0)	35 (5.2)	0.014	0.907
Arthritis	65 (4.6)	33 (4.5)	32 (4.7)	0.045	0.832
Asthma	48 (3.4)	26 (3.5)	22 (3.3)	0.089	0.766
Pain	8 (0.6)	4 (0.5)	4 (0.6)	0.014	0.907
Severity of depressive symptoms, mean (SD)					
HAM-D total 21-item at study entry (*n* = 1407)	19.8 (9.1)	19.2 (9.0)	20.4 (9.0)	0.760	0**.**383
MADRS total at study entry (cMADRS) (*n* = 1409)	24.6 (11.3)	23.2 (11.4)	26.1 (11.0)	14.967	**< 0.001**
MADRS total at onset of the current MDE (rMADRS) (*n* = 1395)	34.1 (7.7)	33.1 (7.7)	35.1 (7.6)	17.226	**< 0.001**
Treatment outcome, *n* (%)^b^					
Response	346 (24.5)	209 (28.5)	137 (20.3)	22.451	**< 0.001**
Non-response	492 (34.9)	268 (36.5)	224 (33.1)		
Resistance	572 (40.6)	257 (35.0)	315 (46.6)		
MADRS total score change (rMADRS–cMADRS), mean (SD) (*n* = 1394)	− 9.4 (10.8)	− 9.7 (10.9)	− 9.0 (10.7)	1.109	0.292
Ongoing additional psychotherapy, *n* (%) (* n* = 1279)	399 (31.2)	183 (27.1)	216 (35.8)	11.113	**< 0.001**
Ongoing psychopharmacotherapy					
Number of concurrently administered psychopharmacotherapeutics, mean (SD)	2.2 (1.2)	2.0 (1.2)	2.4 (1.3)	21.610	**< 0.001**
Daily doses of the first-line AD treatment given in fluoxetine equivalents^c^, mean (SD), mg/day (*n* = 1247)	39.9 (20.8)	40.0 (20.5)	39.6 (21.1)	0.708	0.400
Employed psychopharmacotherapeutic combination and augmentation strategies (in addition to the ongoing AD treatment), *n* (%)					
Any combination and augmentation treatment	855 (60.6)	402 (54.8)	453 (67.0)	22.101	**< 0.001**
Combination with at least 1 additional AD	416 (29.5)	176 (24.0)	240 (35.5)	22.472	**< 0.001**
Augmentation with at least 1 AP	362 (25.7)	159 (21.7)	203 (30.0)	12.912	**< 0.001**
Augmentation with at least 1 MS	159 (11.3)	71 (9.7)	88 (13.0)	3.935	0.047
Augmentation with pregabalin	102 (7.2)	46 (6.3)	56 (8.3)	2.133	0.144
Augmentation with at least 1 low-potency AP^d^	91 (6.5)	35 (4.8)	56 (8.3)	7.204	0.007
Augmentation with benzodiazepines including zolpidem and zopiclone	466 (33.0)	221 (30.1)	245 (36.2)	5.983	0.014

*ADs* antidepressants, *ANCOVA* analysis of covariance, *AP* antipsychotics, *GSRD* The European Group for the Study of Resistant Depression, *HAM-D* Hamilton Depression Rating Scale, *MADRS* Montgomery Åsberg Depression Rating Scale (*cMADRS* current MADRS; *rMADRS* retrospective MADRS), *MDD* major depressive disorder, *MDE* major depressive episode, *MS* mood stabilizer, *n* number of participants, *SD* standard deviation, *SSRIs* selective serotonin reuptake inhibitors

The *p* values displayed in bold were significant after Bonferroni correction

^a^The presence of the current suicidal risk was measured according to the item 3 of the HAM-D rating scale focusing exclusively on suicidality and its extent. Hereby, the absence of the current suicidal risk was reflected by the item-score of 0 (absent), whereas the presence of the current suicidal risk was differentiated by item-scores of 1 (feels life is not worth living), 2 (wishes to be dead or any thoughts of possible death to self), 3 (suicide ideas or gestures) and 4 (suicide attempts)

^b^Non-response was defined by a previous single failed AD trial administered in adequate duration and daily dosing, while treatment resistance was characterized by two or more failed adequate AD trials [3]

^c^AD daily doses were calculated according to Fluoxetine dose equivalents as suggested by Hayasaka and colleagues [3, 32]

^d^Low-potency APs include the so-called low-potency first-generation APs and the second-generation AP quetiapine administered in a daily dose < 100 mg [3]

**Table 2:** Post hoc binary logistic regression analyses investigating the association between the administered first-line AD treatment with SSRIs and parameters identified as significant in our initial analyses in 1410 MDD patients

MDD patients’ characteristics	Adjusted OR (95% CI)/ B ± SE	*p* value
Occupation	0.680 (0.549–0.841)	** < 0.001**
Additional melancholic features	1.530 (1.220–1.918)	** < 0.001**
Current suicidal risk (dichotomous)^a^	1.749 (1.411–2.168)	** < 0.001**
Treatment setting	2.168 (1.708–2.752)	** < 0.001**
MADRS total at study entry (cMADRS)	− 0.019 ± 0.005	** < 0.001**
MADRS total at onset of the current MDE (rMADRS)	− 0.030 ± 0.007	** < 0.001**
Treatment outcome	1.371 (1.073–1.752)	0.012
Additional psychotherapy	1.339 (1.049–1.709)	0.019
Number of concurrently administered psychopharmacotherapeutics	− 0.207 ± 0.045	** < 0.001**
Any combination and augmentation treatment	1.632 (1.312–2.030)	** < 0.001**
Combination with at least 1 additional AD agent	1.565 (1.234–1.986)	** < 0.001**

Table 2 displays results of our *post-hoc* binary logistic regression analyses on the association between the administered first-line AD treatment with SSRIs and variables identified as significant in our primary analyses. These binary logistic regression analyses were adjusted for the variable research center. The *p* values displayed in bold were significant after Bonferroni correction. Adjusted ORs with 95% CIs are presented for dichotomous independent variables, while Bs with SEs are presented for continuous independent variables

*AD* antidepressant, *B* regression coefficient, *CI* confidence interval, *HAM-D* Hamilton Depression Rating Scale, *MADRS* Montgomery Åsberg Depression Rating Scale (*cMADRS* current MADRS, *rMADRS* retrospective MADRS), *MDD* major depressive disorder, *MDE* major depressive episode, *OR* odds ratio, *SE* standard error, *SSRIs* selective serotonin reuptake inhibitors

^a^The presence of the current suicidal risk was measured according to the item 3 of the HAM-D rating scale focusing exclusively on suicidality and its extent [17]. Hereby, the absence of the current suicidal risk was reflected by the item-score of 0 (absent), whereas the presence of the current suicidal risk was differentiated by item-scores of 1 (feels life is not worth living), 2 (wishes to be dead or any thoughts of possible death to self), 3 (suicide ideas or gestures) and 4 (suicide attempts)

## Results

**Fig. 1:**
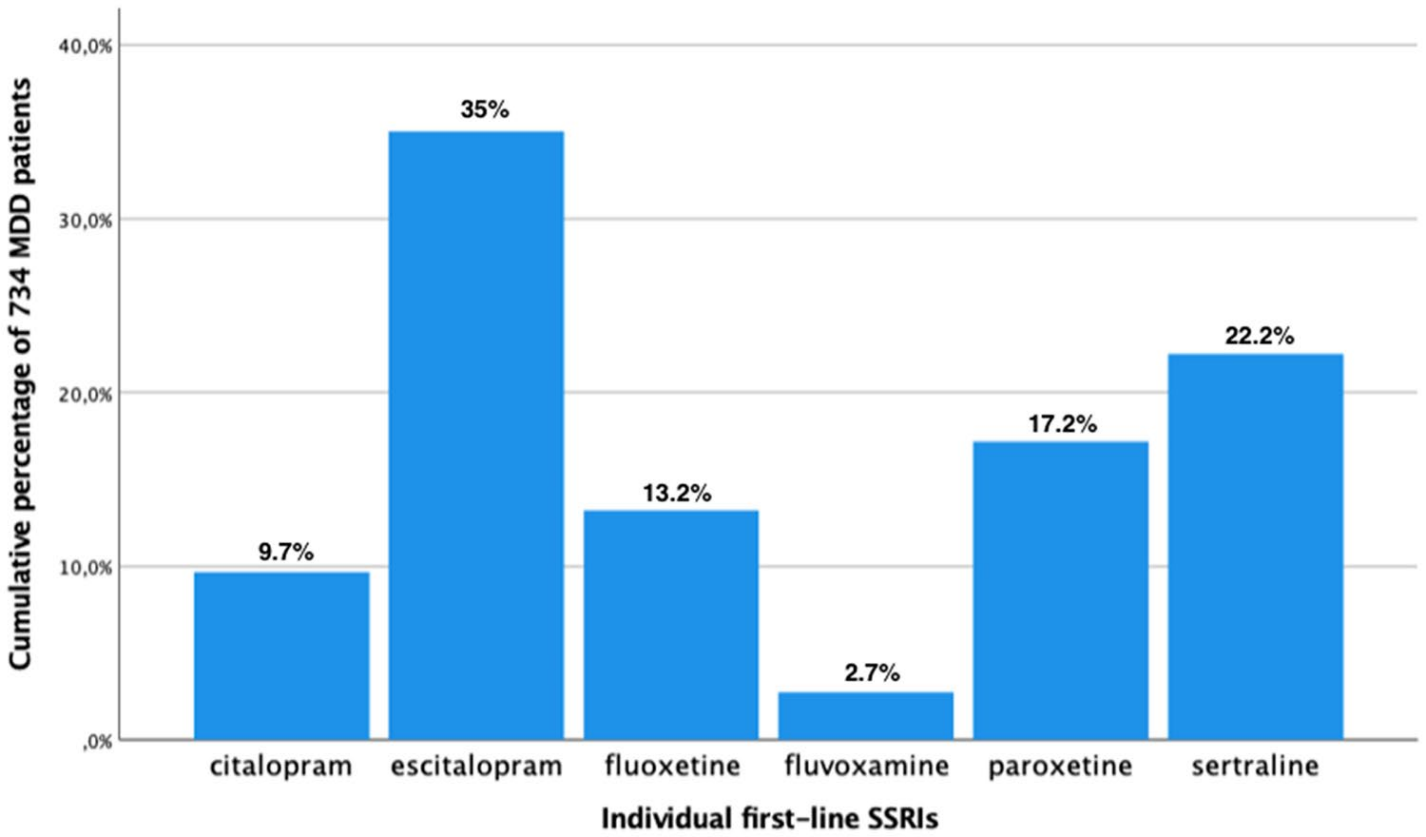
Individual substances administered in 734 MDD patients treated with SSRIs as their first-line AD treatment. Displayed cumulative percentages refer to the individual SSRIs administered as first-line AD treatment in 734 MDD patients. *AD* antidepressant, *MDD* major depressive disorder, *SSRIs* selective serotonin reuptake inhibitors

The total sample included 1410 MDD patients [3] who were treated with either SSRIs (*n* = 734, 52.1%) or other AD substances (*n* = 676, 47.9%) including SNRIs (*n* = 336), noradrenergic-dopamine reuptake inhibitors (NDRIs; *n* = 32), NaSSAs (*n* = 121), serotonin antagonist and reuptake inhibitors (SARIs; *n* = 28), noradrenaline reuptake inhibitors (NARIs; *n* = 3), TCAs (*n* = 74), monoamine oxidase inhibitors (MAOIs; *n* = 5), agomelatine (*n* = 69), tianeptine (*n* = 2), and vortioxetine (*n* = 6) as their first-line AD psychopharmacotherapy during their current MDE. With respect to the individual SSRIs, all six existing substances were distributed in our MDD patients, whereby the majority received escitalopram (*n* = 257) that was followed by sertraline (*n* = 163), paroxetine (*n* = 126), fluoxetine (*n* = 97), citalopram (*n* = 71), and fluvoxamine (*n* = 20; Fig. 1).

The socio-demographic and clinical patterns of the whole sample were comprehensively described in our previous reports and are shown in Table 1 [3]. Table 1 further displays the respective characteristics of the two patient groups split according to their first-line treatment with either SSRIs or other AD substances, and the identified between-group differences. The results of our post hoc binary logistic regression analyses reflecting the association between the administered first-line AD treatment and parameters for which significant between-group contrasts were observed in our initial analyses are depicted in Table 2. Exclusively robust statistical parameters derived from our initial analyses are provided below (*p* values remaining significant in our initial analyses after our correction for multiple testing).

**Fig. 2:**
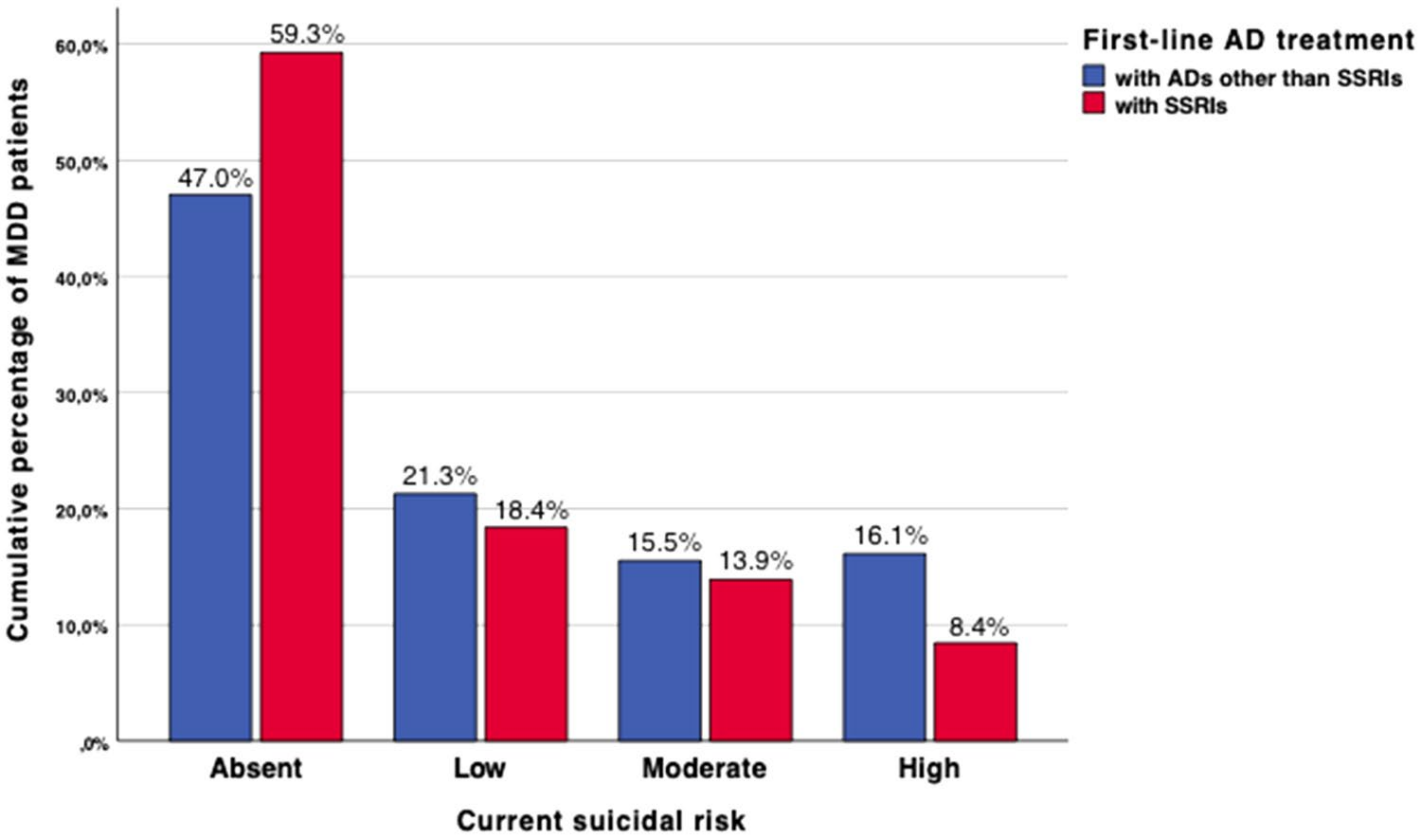
The current suicidal risk of MDD patients receiving either SSRIs or other substances as their first-line AD treatment. Displayed cumulative percentages refer to the proportion of MDD patients receiving either SSRIs (*n* = 737; 52.1%; red colored) or alternative substances (*n* = 676; 47.9%; blue colored) as their first-line AD treatment itemized according to the current suicidal risk and its extent that were ascertained according to the HAM-D item 3 that is exclusively dedicated to suicidality [17]. While the absence of the current suicidal risk was reflected by the item-score of 0 (absent), its presence was represented by item-scores of 1 (feels life is not worth living), 2 (wishes to be dead or any thoughts of possible death to self), 3 (suicide ideas or gestures) or 4 (suicide attempts). While significant between-group differences were detected in terms of the presence of the current suicidal risk (*p* < 0.001), MDD patients receiving first-line SSRIs did not significantly differ from their counterparts with respect to its extent (*p* = 0.214). *AD* antidepressant, *HAM-D* Hamilton Depression Rating Scale, *MDD* major depressive disorder, SSRIs = selective serotonin reuptake inhibitors

**Fig. 3:**
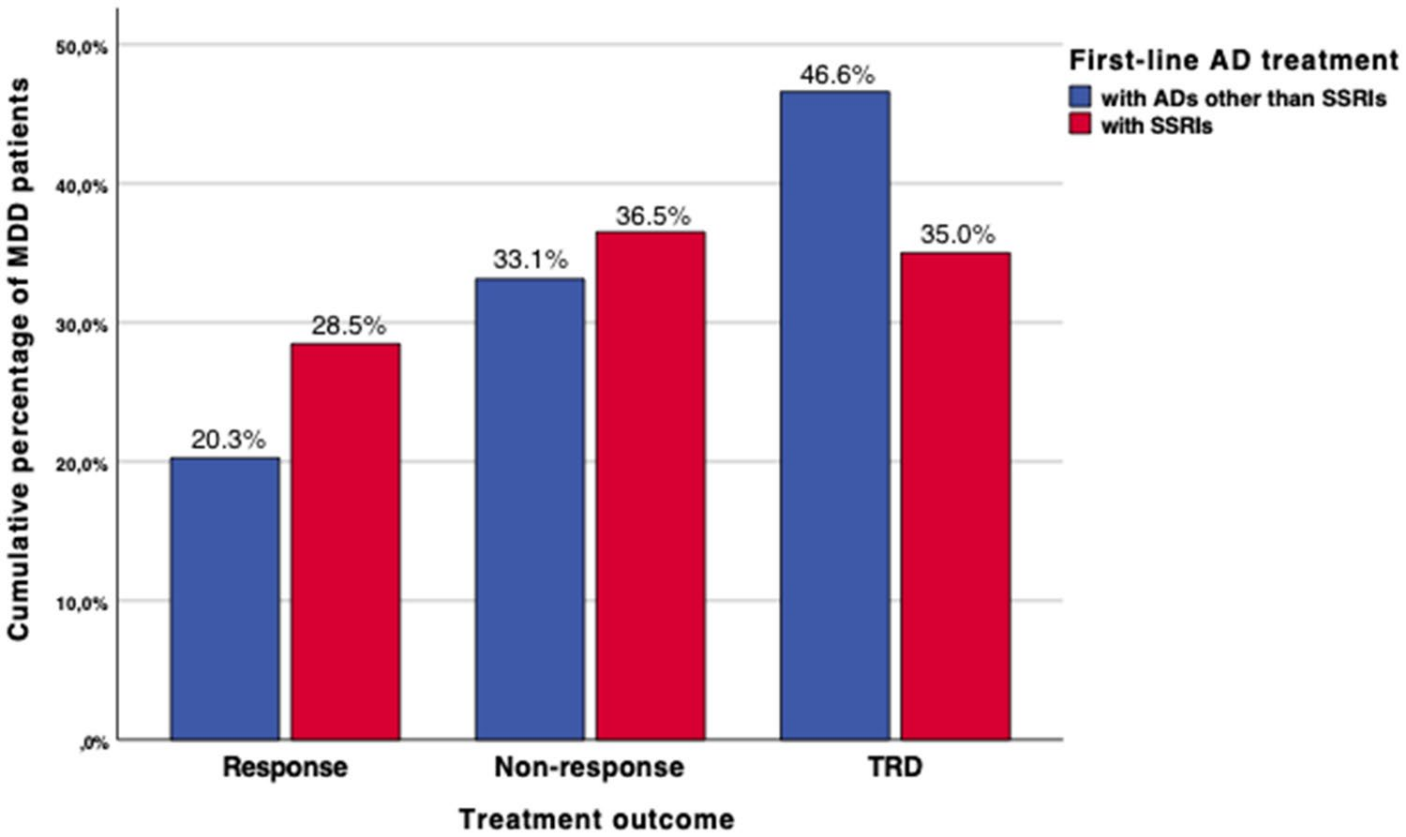
Treatment outcome patterns in MDD patients receiving either SSRIs or other substances as their first-line AD treatment. Displayed cumulative percentages refer to the proportion of MDD patients receiving either SSRIs (*n* = 737; 52.1%; red colored) or alternative substances (*n* = 676; 47.9%; blue colored) as their first-line AD treatment itemized according to their treatment outcome patterns reflecting response, non-response and TRD that differed significantly between both patient groups in our initial analyses (*p* < 0.001). While non-response was defined by a previous single failed AD trial, at least two failed AD trials were mandatory for TRD. *AD *antidepressant; *MDD *major depressive disorder; *SSRIs* selective serotonin reuptake inhibitors, *TRD* treatment resistant depression

MDD patients who underwent first-line AD treatment with SSRIs during their current MDE showed lower odds for unemployment as compared to their counterparts receiving other agents (48.4% vs 58.4%, *p* < 0.001). The current occurrence of melancholic features (54.5% vs 67.5%, *p* < 0.001) and suicidal risk (40.1% vs 52.5%, *p* < 0.001; Fig. 2) was less frequent in patients treated with first-line SSRIs in relation to those with other substances. Patients receiving SSRIs were less frequently treated as inpatients during their current MDE (25.2% vs 44.8%, *p* < 0.001). The severity of depressive symptoms measured with the MADRS at onset of the current MDE (mean rMADRS total score 33.1 ± 7.7 vs 35.1 ± 7.6, *p* < 0.001) as well as at study entry (mean cMADRS total score 23.2 ± 11.4 vs 26.1 ± 11.0, *p* < 0.001) was lower in this subgroup than in patients receiving alternative agents. While response to the first-line AD treatment occurred more frequently in patients treated with SSRIs (28.5% vs 20.3%), TRD was diagnosed more commonly in patients with alternative first-line AD psychopharmacotherapy (35.0% vs 46.6%, *p* < 0.001; Fig. 3).

In terms of the administered therapeutic strategies during the current MDE, patients taking SSRIs received a lower mean number of psychopharmacotherapeutics (2.0 ± 1.2 vs 2.4 ± 1.3, *p* < 0.001) and underwent additional psychotherapy (27.1% vs 35.8%, *p* < 0.001) less frequently than patients treated with other first-line ADs. Psychopharmacotherapeutic augmentation and/or combination strategies in general (54.8% vs 67.0%, *p* < 0.001) and augmentations with at least one antipsychotic (AP) agent (21.7% vs 30.0%, *p* < 0.001), and combination treatment with at least one additional AD (24.0% vs 35.5%, *p* < 0.001) in particular were less commonly prescribed in patients treated with first-line SSRIs as compared to their counterparts.

The aforementioned between-group contrasts remained significant in our post hoc binary logistic regression analyses with exception of the associations between first-line AD treatment with SSRIs and treatment outcome (*p* = 0.012) as well as employment of additional psychotherapy (*p* = 0.019; Table 2).

## Discussion

In the present multinational and naturalistic cross-sectional secondary investigation, about half of the included MDD patients (52.1%) received SSRIs as their first-line AD treatment during their current MDE, while the remaining group was treated with alternative ADs. Patients receiving SSRIs exhibited a favorable socio-demographic and clinical profile with reduced odds for unemployment and for additional features occurring during the current MDE such as melancholia and suicidality. Furthermore, they also showed reduced odds for current inpatient treatment and for additional therapeutic strategies including combination with other ADs and augmentation with APs. Importantly, patients undergoing first-line AD treatment with SSRIs exhibited a lower severity of depressive symptoms at the onset of the current MDE as well as at study inclusion. A trend towards higher rates of treatment response was observed in patients with SSRIs, while treatment resistance occurred trend-wise more commonly in patients taking alternative first-line ADs.

Our findings of preferred first-line treatment with ADs other than SSRIs in melancholic depression, that are in line with available international evidence, may reflect common clinical prescription practice in a severe MDD subtype with specific clinical manifestations and related neurobiological correlates [16, 52]. Specifically, patients with melancholia were previously shown to respond less to placebo and psychotherapeutic and psychosocial treatments [61], whereas rapid and better outcomes following biological treatments comprising ADs or electroconvulsive therapy were observed [60, 62]. In fact, a lately published meta-analytical report revealed that patients with melancholia in MDD appear to achieve a greater reduction of their symptoms with antidepressant treatment but also with placebo compared to those lacking these features [38]. Further, recent evidence reported differential response rates among several classes of ADs, whereby SSRIs seemed to be less efficacious than other ADs, especially TCAs [70, 71]. With respect to current treatment guidelines, exclusively the American Psychiatric Association specifically advises TCAs and SNRIs in the treatment of melancholic depression in their CPGs [25].

The lower proportion of SSRI prescriptions in MDD patients exhibiting current suicide risk is worth mentioning, since suicidality represents a major burden in MDD [39] that must be considered and regularly assessed in each patient, and since warnings on the provocation of suicidality with SSRIs were propagated previously. The question of AD agents including SSRIs and suicide risk in clinical trials has been subject to a vigorous scientific debate with disparate results and conclusions that often yielded from differential methodological analyses of the same data sets [33, 34, 40, 47]. Importantly, epidemiological data clearly displayed a reduction of suicides parallel to an increase of prescriptions of ADs and might, thus, more likely reflect the real-world situation of the respective risk inherent to this type of psychopharmacotherapy [14]. Hereby, the latter study results highlight that suicidality represents one of the most frequent and serious symptoms occurring during MDEs per se rather than a treatment-related consequence. Our data might be a demonstration that clinicians are reluctant to primarily prescribe SSRIs in patients who exhibit suicidal thoughts or ideations because of the exemplified controversial scientific discussion that used to be extensively reported in mainstream, non-scientific media. It is a well-known phenomenon that ADs acting via the reuptake inhibition of serotonin bear the potential to induce agitation or restlessness after their initiation [8]. It is, however, noteworthy in this context that the redoubtable related suicidal behavior is very well avoidable with adequate therapeutic strategies including co-administration of agents with tranquilizing effects and/or inpatient treatment setting and might be, hence, of clinical relevance exclusively in case of inadequate treatment [1, 5, 17, 57, 67].

The observed lower prescription rates of SSRIs in suicidal MDD patients might as well refer to the fact that suicidality was repeatedly associated with chronicity as well as treatment resistant depression (TRD) and/or difficult-to-treat depression (DTD) [3, 17, 54] representing conditions, where AD agents with different mode of action, such as the MAOI tranylcypromine for instance, are preferably recommended in international treatment algorithms for MDD [49]. Furthermore, the NaSSA mirtazapine, that showed a faster onset in comparison to other ADs in several studies, was suggested as a noteworthy alternative consideration of initiating a first-line AD especially in MDD patients suffering from suicidality [6, 7, 42]. Even though not evaluated in our trial, the potent AD as well as anti-suicidal effects of esketamine have to be mentioned in this context, as this compound has an important field of application in this indication due to a very rapid AD onset of action [50, 51, 55, 72].

Our further associations between first-line SSRI treatment and a lower severity of depressive symptoms during the current MDE and a lesser need for current and/or previous inpatient treatment support our consensus postulating that a rather favorable disease profile may serve as a possible variable guiding clinicians’ treatment choice towards SSRIs. Another possible reason for the observed prescription rationale may have derived from evidence postulating a varying effectiveness of SSRIs in dependence of the severity of depressive symptoms [59]. Importantly, while a negative relationship between baseline severity of depressive symptoms and response to SSRIs was found in meta-analyses by some authors [23], others came to contrary conclusions [22]. Most recently, one of the largest and comprehensive patient-level investigations was able to deliver compelling evidence that SSRIs are efficacious in the whole spectrum from mild to severe depression by applying a different, coherent methodological approach, e.g. an item-based analysis [35].

In terms of treatment outcome, our results revealed a trend towards higher response rates in MDD patients treated with SSRIs as first-line AD treatment, while the prescription of ADs with different modes of action were associated with a trend towards treatment resistance. Being aware of existing international evidence on AD efficacy in MDD favoring alternative AD substances over SSRIs and vice versa [9, 15, 37, 59] as well as individual agents within the SSRI substance class [43], the largest NMAs dedicated to this topic suggested comparable AD potency of over 20 commonly prescribed ADs [11]. However, the highly selective patient populations derived from RCTs and the fact that conclusions from NMA about efficacy and/or tolerability of ADs are not equal to direct head to head comparisons [45] limit their overall explanatory power. Given the cross-sectional nature of our study with retrospective evaluation of treatment outcome, we cannot assume any difference in the efficacy of SSRIs compared to other AD classes with certainty. A possible bias resulting of allocating less severely ill patients to SSRIs rather than other ADs may have affected our results. To sum up, our naturalistic data may primarily represent a valuable contribution revealing a real-world AD prescription culture rather than providing any information about efficacy of the administered compounds.

The significantly reduced odds for add-on treatments including AD combinations and augmentation with APs in MDD patients who received SSRIs might represent complementary results highlighting that a further therapeutic escalation is inevitable in case of insufficient response to first-line ADs, that in general affects a considerable number of MDD patients worldwide [3, 5, 18, 48, 49] and that was more commonly encountered in our patients receiving alternative first-line substances. The trend-wise less frequently employed additional psychotherapies might go along with the fact that patients who achieve response or even remission under their first-line AD treatment, which consists of SSRIs in the most cases [5, 19, 64], more likely forgo additional psychotherapy for various reasons [4, 28].

Higher rates of unemployment in MDD patients receiving first-line ADs other than SSRIs represented our only significant between-group contrast in terms of socio-demographic aspects and was mostly interpreted in the context of illness severity as well as functional impairment that was more pronounced in our patients with alternative first-line ADs. Precisely, patients showing a lower severity of depressive symptoms as well as less functional impairment may predominantly have been allocated to SSRI first-line medication. The latter assumption might be supported by available international data reporting positive effects of different individual SSRIs and other classes of ADs on workplace functioning [53].

Noteworthy strengths of the present study are the naturalistic design and the large international sample which may best possibly reflect the broad everyday routine. In contrast to the most RCTs, such real-world patient population gathered from in- and outpatient units of university as well as non-academic centers in eight countries allows investigations of heterogeneous clinical manifestations of MDD including suicidality, psychotic features, psychiatric and/or somatic comorbidities, and varying disease course and severity ranging from single to recurrent MDEs with mild, moderate or severe extent of current depressive symptoms. However, potential cross-site differences of the prescription practice, driven by the type of recruiting institution (academic vs. non-academic), divergent insurance situations as well as availabilities and approvals of the specific psychopharmacotherapeutics which might have arisen by recruitments in different European countries, cannot be fully ruled out. To minimize a distortion of our result in these regards, the variable “research center” was accounted for in our statistical analyses. Furthermore, physician-related factors as exact number of years of their experience, which may have influenced treatment patterns and the findings, respectively, were not systematically assessed. To minimize potential biases related to the latter aspects and to assure a high standard of data quality and inter-rater reliability, exclusively experienced psychiatrists undergoing specific rater trainings were allowed to perform the comprehensive clinical assessments of the enrolled MDD patients who were treated by senior consultants for psychiatry in all recruiting centers. Furthermore, the present large multi-site project conducted by the GSRD [3, 66, 69] was primarily designed to elucidate clinical and genetic aspects of TRD, whereby the current secondary analyses focusing on the first-line AD treatment and the related socio-demographic and clinical characteristics represent an additional aspect which bears potential limitations.

Furthermore, the open treatment design may be subject to bias regarding assessment and allocation. The fact that about a half of the included 1410 MDD patients received SSRIs as first-line psychopharmacotherapy and a comparable group of patients was treated with other ADs bears the limitation of relatively small proportions of AD agents that were individually prescribed in the group of patients receiving alternative ADs. This led us not to differentiate between the distinct individual ADs to enable investigations of comparable groups of MDD patients. Similarly, we did not differentiate between the individual SSRIs, which we deem justifiable in light of the fact that superiority of a specific substance could not be demonstrated with certainty and due to the unequal proportion of MDD patients treated with the respective individual SSRIs. While exclusively conventional on-label substances were involved in the current investigation, novel psychopharmacotherapeutic options that have recently been shown to be very effective and, hence, approved in MDD and/or TRD, as esketamine for instance [41, 44, 49, 65], have not yet been considered. Furthermore, it is noteworthy that the psychopharmacotherapeutic terminology applied in our work is based on the traditional indication-based nomenclature to ensure an unhampered interpretability and comparison to available international literature, even though we are very well aware of a new classification system that is increasingly replacing the current terminology. The so-called Neuroscience-based Nomenclature (NbN) is driven by the pharmacological profiles of the individual substances and is, hence, thought to support rational and lucid prescribing with the goal to increase therapeutic adherence of the patients [24, 74].

Most importantly, it has to be pointed out that the cross-sectional design of the study does not allow to ascertain any causal conclusions and, hence, represents an explicit limitation. Clearly acknowledging that this procedure yields less accurate results than prospective investigations, we would like to highlight our treatment outcome measures that were calculated according to the total score reduction between the rMADRS, referring to a time-point when the depressive symptoms reached their maximum (minimally 4 weeks prior inclusion), and the cMADRS, representing a time-point of study entry (at least after four weeks of an adequate psychopharmacotherapy). The respective variables reflecting a reduction of depressive symptoms during the current MDE might be, hence, regarded as longitudinal measures providing hints towards causality. We are aware that this approach is inferior to randomized-controlled, prospective conditions; however, in light of the fact that MDD patients were previously shown to adequately recall symptoms for a considerable period of time [20] and that many rating scales consider symptoms of MDD retrospectively, we deem our procedure justifiable. Additionally, to minimize such associated bias, all our raters were experienced psychiatrists undergoing extensive training in the respective scales.

## Conclusion

The observed beneficial socio-demographic and clinical profile associated with first-line SSRI administration in contrast to the rather inferior characteristics related to alternative substances may reflect broad adherence of European psychiatrists to the current international treatment algorithms suggesting SSRIs for the initial treatment approach in MDD, while AD substance classes like MAOIs or TCAs are recommended once sufficient treatment response could not be achieved [49]. Furthermore, clinicians may deem SSRIs less appropriate in treating psychopathological features as suicidality and/or melancholia, which may partly result from previous evidence reflecting conflicting or ambiguous findings. The fact that the abovementioned contrasts between patients receiving SSRIs and other substances were identified in the course of a cross-sectional retrospective study with an open selection of either treatment leads us to interpret them as very useful variables to understand the criteria guiding the choice of the first-line AD in MDD in real-world settings, rather than they may reflect any relevant pharmacological differences in mechanisms of action of the investigated ADs.
